# Supplementary material for: Oral Bioavailability and Metabolism of Hydroxytyrosol from Food Supplements
Source: Nutrients. 2023 Jan 9;15(2):325. doi: 10.3390/nu15020325 (PMC9866489; doi:10.3390/nu15020325)
Supplement: Supplementary file 1 [file nutrients-15-00325-s001.zip › nutrients-2079747-supplementary.pdf]

Table S1: Selection criteria for volunteers.

**Inclusion Criteria**	Self-reported healthy men aged 21 - 50,<br>·subject had an adequate understanding of the study and signed the informed consent to participate in the study,<br>·willingness to follow dietary and physical activity restrictions during study participation,<br>body mass index range: >18.5 and <29.9 kg/m^2,<br>sex eligible for study: male,<br>·accepted healthy volunteers
**Exclusion Criteria**	Any known allergies to IPs (olives and their derivates),<br>·any acute or chronic diseases (e.g., diagnosis of diabetes mellitus, hypertension, dyslipidemia, or other cardiometabolic disorders; diagnosed hepatic, renal, or cardiovascular disease),<br>any kind of eating disorders,<br>not fluent in German,<br>any previous (last 14 days prior to screening) or ongoing pharmacological therapy (e.g., any medication, vaccination, infusion),<br>·any intake of nutritional supplements,<br>any known addiction to drugs and/or alcohol,<br>smoker,<br>investigator or physician doubts truthfulness of self-reported health information
